# Association between Phthalate Exposure and Frailty among Community-Dwelling Older Adults: A Repeated Panel Data Study

**DOI:** 10.3390/ijerph18041985

**Published:** 2021-02-18

**Authors:** Hongsoo Kim, Seyune Lee, Young-Il Jung, Yun-Chul Hong

**Affiliations:** 1 Graduate School of Public Health, Department of Public Health Sciences, Seoul National University, Seoul 08826, Korea; hk65@snu.ac.kr (H.K.); seyunel@gmail.com (S.L.); 2 Institute of Health and Environment, Seoul National University, Seoul 08826, Korea; 3 Institute of Aging, Seoul National University, Seoul 08826, Korea; 4 Department of Environmental Health, Korea National Open University, 86 Dongsung-Dong, Jongno-gu, Seoul 03087, Korea; extra012@knou.ac.kr; 5 Department of Preventive Medicine, Seoul National University College of Medicine, 103 Daehakno, Jongrogu, Seoul 03080, Korea

**Keywords:** healthy aging, environmental pollutants, nutrition, community, Asia

## Abstract

Only a few studies have examined the impacts of environmental exposure on frailty. This study investigated the association between phthalates and frailty among community-dwelling older adults. The Korean Elderly Environmental Panel II (KEEP II) study is a repeated panel data study of 800 community-dwelling older adults in South Korea. Frailty was measured with five items defined by Fried and colleagues. Environmental pollutants in the form of two types of metabolites for Di-ethylhexyl phthalate (DEHPs)—Mono (2-ethyl-5-hydroxyhexyl) phthalate (MEHHP) and Mono (2-ethyl-5-oxohexyl) phthalate (MEOHP)—were obtained from urine specimens. Analyses were performed using repeated linear mixed models. The concentration levels of both MEOHP and MEHHP in urine were significantly higher in the pre-frail or frail group than its counterparts. While adjusting for covariates, MEOHP level was positively associated with the likelihood of being pre-frail or frail in both males and females; the concentration level of MEHHP also had a positive impact on the likelihood of being pre-frail or frail in females. The DEHP metabolite concentrations were significantly lower among adults with daily fruit consumption in both males and females. DEHPs, measured by metabolite concentrations, may increase the risk of frailty among older men and women; further studies are necessary. The preventive effects of nutrition on DEHP risk should also be further investigated.

## 1. Introduction

Global aging has accelerated, and countries have been paying more attention to strategies to promote healthy aging [1]. Frailty refers not to disease or disability but pathological conditions that can cause various symptoms and was first addressed by the Federal Council on Aging [2]. As a clinical syndrome that can exacerbate physiological vulnerability, frailty is known to be a precursor to serious disability and disease, but it can be prevented and delayed [3,4]. The syndrome indicates older adults are at a high risk for functional aggravation, and noticing it allows room to intervene to prevent and/or delay the onset of such irreversible functional decline, unlike disease and disability [3,4,5].

Frailty is complex, but there has been pretty consistent evidence in the literature across countries on its socio-demographic and behavioral determinants. Researchers have investigated a wide variety of factors associated with frailty, including socio-demographic characteristics, marital status, social support, experience of fall, number of comorbid diseases, and medications [3,6]. Yet there are few examined environmental determinants of frailty. The environmental health literature has identified older adults as a group highly susceptible to environmental exposure [7], and researchers have recommended further investigations on high-risk groups among the older population [8,9]. Environmental health studies mainly have reported empirical evidence on the relationships between specific exposures and certain diseases or mortality [10]. However, limited studies have examined the impacts of environmental exposure on frailty, which is the gap this study is intended to address.

The U.S. Environmental Protection Agency (U.S. EPA) has proposed a research framework for aging and the environment with consideration to the diversity of health and function in later life; the framework posits potential interactions between environmental exposure, health, and additional sources of variability with aging [7]. Factors such as gender, socioeconomic status (SES), lifestyle, nutrition, and region of residence characterize the older population, and these can also be determinants of environmental exposure and health outcomes [7,11,12]. Therefore, guided by the EPA’s framework, this study aimed to examine the impact of environmental exposure—in particular, phthalate exposure—on frailty using longitudinal observation data.

Phthalates including 2-ethylhexyl phthalates (DEHPs) are widely used industrial chemicals, and they are included in a variety of quotidian products, such as food packaging, cosmetics, personal care products, plastics, furniture, and building materials [13,14,15]. A large number of studies have reported associations between DEHPs and diverse health effects such as allergies, asthma, and chronic inflammation [13,15,16,17,18]. Recent studies have also found a positive association between levels of DEHP concentration and lung function, handgrip strength, and even depressive symptoms in Korean older adults [19,20,21]. The objective of this cohort study was to investigate the association between DEHP exposure and frailty in older adults aged 60 or older in South Korea, an East Asian country with the most rapidly aging population in the world [22]. At the time of this writing, we had found no published study examining the impacts of DEHP exposure on frailty.

## 2. Materials and Methods

### 2.1. Study Settings and Sample

The Korean Elderly Environmental Panel II (KEEP II) study aimed to examine the associations between environmental risk factors and health outcomes among older adults aged 60 or older in South Korea. The KEEP study sought to identify potential environmental risk factors for various adverse health effects in the older population and to suggest risk communication strategies for vulnerable groups in Korea. As environmental risk factors, we measured heavy metals (Pb, Hg, and Cd), endocrine disruptors (phthalate metabolites), and Pyrethroid (3-PBA). We also examined several health outcomes including heart rate variability, pulmonary function, cognitive function, depression, and frailty.

The study panel began with a total of 800 older people in two regions. The inclusion criteria of the sample were (1) adults aged 60 or older, (2) who were residing in a community, and (3) who were capable of communicating and participating in a physical examination. No exclusion criteria were specified for the study, which aimed to recruit a wide range of non-institutionalized, community-dwelling older adults.

The KEEP II, as a three-year repeated measures study, was conducted between September 2012 and May 2015. We collected data up to three times for each participant in three waves during the winter season (November–March) to reduce potential seasonal effects. When participants dropped out, we invited new ones, and thus the panel size was kept to 800 for both the second and third waves. The participants enrolled later as replacements for drop-out cases had measurements of their own, not replacing the measurements of the drop-out cases. Data collection using a standardized study protocol consisted of obtaining blood and urine specimens, physical and functional assessments, and a structured interview survey.

The KEEP II research team developed a survey questionnaire that comprised a wide range of questions on topics including demographics, SES, health behaviors, diets, environmental exposure-related questions, medical history, and health status (e.g., comorbidity, cognitive function, depression). Some of the health behavior and diet-related items were adopted and modified from the Korean National Health and Nutrition Examination Survey (KNHANES), a nationwide survey that examines the health and nutritional status of a representative sample of Koreans. The team conducted physical and functional assessments (e.g., anthropometric measurement, handgrip strength, walking speed) and collected and analyzed urine and blood specimens. The details of the KEEP II study have been reported elsewhere [20,21]. The Institutional Review Board at Seoul National University Hospital approved the study protocol (C-1209-006-424).

In this specific study, we used survey data, urinary phthalate concentration, and physical assessment data to determine frailty status. The analyses included a total of 767 cases at the baseline (first wave) without missing values in the key variables of the study, with 748 and 771 in the second and third waves, respectively. The study sample, an unbalanced panel allowing new recruitment for dropouts, consisted of a total of 2286 cases (observations) from 1228 unique participants over the three measurement points. There were 361 and 100 new participants in the second- and third-wave surveys, respectively.

### 2.2. Measures

Frailty, the outcome variable, was measured using the five clinical conditions defined by Fried et al.’s [3] study as a phenotype of frailty in community-dwelling older adults. The five criteria (items) were shrinking (unintended weight loss), weakness (a decrease in the strength of handgrip), poor endurance (self-reported severe fatigue), slowness (a decrease in walking speed), and low physical activity (a decrease in activity amount, kcal/week). Based on the scores of the five items, we categorized participants into three groups according to the number of applicable conditions: frail (3+), pre-frail (1–2), and fit (no conditions). The cut-off points in the ”weakness”, “slowness”, and “low physical activity” items were defined using a national survey of Koreans aged 60 or older administered by the Ministry of Health and Welfare [23]. The criteria, items, and measures of frailty in this study are summarized in Table 1.

The research team provided training sessions for the data collection of frailty measures. Handgrip strength was measured using a grip dynamometer (Hand Grip Meter 6103, Tanita, Tokyo, Japan). The trained data collectors first demonstrated to the older adults how to use the dynamometer with an explanation that the participant’s shoulder must be adducted and the elbow should be flexed at 90 degrees. Study participants were asked to measure both their right and left hand twice each with a one-minute interval between measurements, and the highest value was used to produce the frailty index. Walking speed was also measured twice, and the lower value of the two was used. Fatigue was based on a self-reported survey item. Weight and height were directly measured with an electrical measuring machine (and a tapeline was used for those who were severely bent).

Phthalate exposure, the key explanatory variable, was measured using a urine sample we collected to evaluate the level of two types of metabolites of 2-ethylhexyl phthalate (DEHP): mono-(2-ethyl-5-hydroxylhexyl) phthalate (MEHHP) and mono-(2-ethyl-5-oxohexyl) phthalate (MEOHP). Our team collected a spot urine sample of about 20 mL (mid-stream urine) from each participant between 9 a.m. and 11 a.m. on the day of each test. We stored the sample immediately in a freezer at −20° Celsius until analysis, which was within one month of collection. Data collectors received education and training on collecting, handling, delivering, and storing the specimen according to a standardized protocol. Our analysts preprocessed and analyzed the urinary phthalate metabolites using a high-performance liquid chromatography system (HPLC, Nexera X2 system, SHIMADZU, Kyoto, Japan), based on previously reported standard procedures [24].

We also collected data on the socio-demographic, lifestyle, and health conditions of participants through an interview survey. We selected these covariates based on an extensive literature review and also findings from other studies using the KEEP II data. Socio-demographic data included age, gender, education, household type, house income, occupation, and living location [3,5,6,9,10,11,12,19,20,21]. Lifestyle data included smoking, drinking, and fruit and vegetable consumption [6,10,12,19,20,21,25]. “Non-smoker” refers to a person who reported smoking fewer than 20 packs of cigarettes during their lifetime, and “non-drinker” refers to a person who reported (s)he could not or did not drink alcohol (e.g., due to religion). For fruit and vegetable consumption, participants were categorized into two groups according to whether or not they had fruit and/or vegetables every day, based on the Korean Dietetic Association’s guidelines for older adults [26]. In order to adjust for health conditions, we included in the analysis body mass index (BMI; kg/m^2^) and a number of chronic diseases (cardiovascular disease, cerebrovascular disease, chronic obstructive pulmonary disease, hypertension, diabetes mellitus, cancer, and arthritis). We categorized BMI into three groups (below 23, 23–25, and above 25) according to clinical practice guidelines for overweight/obesity status in Korea [27].

### 2.3. Statistical Analysis

We computed descriptive statistics using a t-test, Chi-square test, and analysis of variance (ANOVA) with the baseline data in order to obtain the frequency and distribution of the socio-demographic, lifestyle, and health conditions of older adults by frailty and also to examine the relationships between DEHP concentration and frailty status. Due to skewness, urinary DEHP metabolites were presented as geometric means and transformed using the logarithm function [21].

We applied generalized estimating equations (GEEs) to the panel data in order to find out the association between each DEHP metabolite and frailty. We used mixed modeling methods with a variance-covariance matrix in the panel data analyses in order to assess the associations between socio-demographic, behavioral, and health conditions and each DEHP metabolite. We conducted all statistical analyses using SAS version 9.4 (SAS Institute Inc., Cary, NC, USA).

## 3. Results

Table 2 shows the general characteristics of the study population at baseline by frailty status. In terms of frailty, less than half (42.2%) were fit, while 57.8% were frail (11.5%) or pre-frail (46.3%). The frailty status of older participants was significantly different by socioeconomic position. Those who were older, had lower education levels, and/or had lower household income were more likely to be pre-frail or frail. In addition, older adults who were working and/or resided in rural areas tended to be pre-frail or frail. As for dietary behaviors, older adults who consumed fruits and vegetables every day were less likely to be pre-frail or frail. Health condition-related variables were also associated with frailty; those with more drugs, a lower BMI, and/or more chronic diseases were more likely to be frail.

Table 3 shows the concentration levels of creatine-adjusted urinary DEHP metabolites (MEOHP, MEHHP) for fit and non-fit (pre-frail or frail) participants at baseline. The geometric means of creatine-adjusted concentrations were 28.06 μg/L for MEHHP and 21.39 μg/L for MEOHP. Both MEHHP and MEOHP concentrations were significantly lower in the fit group (22.40 μg/L for MEHHP, 17.52 μg/L for MEOHP) than the pre-frail or frail group (33.08 μg/L for MEHHP, 24.75 μg/L for MEOHP). The concentrations of urinary DEHP metabolites in this study sample were relatively high compared to the concentrations of urinary DEHP metabolites in the national environmental health investigation of a nationally representative sample of older adults in 2015–2017 (MEHHP: 21.5 μg/L in age 60–69, 25.6 μg/L in age 70+; MEOHP: 15.8 μg/L in age 60–69, 20.2 μg/L in age 70+) [28]. When the participants were divided into quarters according to DEHP concentrations, the number of people in the first quartile was high in the fit group, and the number of people in the third and fourth quartiles was high in the pre-frail or frail group.

The associations between DEHP metabolite concentrations and frailty of the study population using GEE analyses are presented in Table 4. In the unadjusted model (Model 1), log-transformed MEHHP (OR = 1.58, 95% CI = 1.38–1.81, *p* < 0.001) and MEOHP (OR = 1.51, 95% CI = 1.34–1.71, *p* < 0.001) concentrations in the total sample were positively associated with frailty. The positive relationship between both DEHP metabolite concentrations and frailty was consistent while adjusting for SES only (Model 2); SES and lifestyle (Model 3); and SES, lifestyle, and health conditions (Model 4). QIC—goodness of fit statistics for GEE—was the smallest in Model 4. In the male sub-sample, only MEOHP concentrations increased the likelihood of being pre-frail or frail (OR = 1.33, 95% CI = 1.02–1.73, *p* = 0.037) when all the covariates were adjusted for (Model 4). In the female sub-sample, both MEHHP (OR = 1.28, 95% CI = 1.06–1.55, *p* = 0.012) and MEOHP (OR = 1.20, 95% CI = 1.01–1.43, *p* = 0.037) concentrations had consistently positive impacts on the risk of being pre-frail or frail in Model 4 while adjusting for all the covariates.

Table 5 includes further investigations of the health behavior factors associated with DEHP concentrations. In the multi-year, multivariate panel analyses, older adults having fruit every day had significantly lower MEHHP (β = −0.11, SE = 0.07, *p* = 0.001) and MEOHP (β = −0.10, SE = 0.03, *p* = 0.005) levels. In the subgroup analyses by gender, older men having fruit every day only had a lower level of MEHHP (β = −0.14, SE = 0.07, *p* = 0.043) than their counterparts. Among older women, fruit consumption was negatively associated with the levels of both MEHHP (β = −0.10, SE = 0.04, *p* = 0.006) and MEOHP (β = −0.09, SE = 0.04, *p* = 0.028).

## 4. Discussion

This study empirically demonstrated a significant positive association between DEHP (environmental pollutants) and frailty with a cohort of community-dwelling older adults based on the EPA’s framework on aging and health. We also observed large variations in DEHP concentrations among the older participants in the sample. Older adults can be exposed to DEHP known to be an endocrine-disrupting chemical in their daily living commonly and/or often unconsciously through various routes, including the use of plastic food containers, skin-care products, scent preservatives, and other industries [29,30]. DEHP concentration levels differed between the fit and non-fit (pre-frail or frail) groups, consistent with our multi-stage models and subgroup analyses by gender.

A wide range of studies have been conducted on the socioeconomic and health behavioral factors aggravating and preventing the onset and progress of frailty [6,31,32], and the relationships between environmental agents and frailty have been explored recently [8]: associations between walking speed or grip strength (components of the frailty index) and cotinine concentration, particulate air pollution, or exposure to heavy metals were reported in an older population [20,33,34,35,36,37,38]. However, few empirical studies have assessed the effects of phthalate exposure on frailty, and this is an initial study on the effects of phthalate exposure on the frailty of older adults.

Most existing studies on the health effects of phthalates have focused on children and females of childbearing age [39,40]. This study provides evidence that phthalate exposure is also an important health risk to the older population. The duration of exposure increases with extended life expectancy, and this can result in chronic health effects [41,42]. The aging process is accompanied by declining functional capacity, and the role of the endocrine system is to maintain body function. If chemicals disrupt this system, they may accelerate functional decline, resulting in more serious health effects for the older population. In fact, older adults can easily be exposed to phthalates during their daily living through consuming pre-packaged foods and using medications, nutritional supplements, or medical devices [13]; thus, the findings of this study have public health implications. Further studies on the pathways of phthalate exposure, frailty, and disease are needed, based on which healthy living environment programs and interventions for healthy aging can be developed and implemented.

In terms of outcomes, environmental pollutant (exposure) and health outcome studies conducted on adults mostly have been focused directly on diseases [13,16,17,18]. Frailty has been reported as a precursor to morbidity and disability that can be prevented or delayed through public and individual-level health interventions [4,5]. Thus, the findings of this study on the positive relationship between phthalates and frailty suggest a pathway to reduce the impact of environmental pollutants on diseases through frailty and also imply that, through frailty prevention, we may delay or disconnect the link between environmental pollutants and the onset or aggravation of diseases. Further investigations on the relationships between environmental pollutants, frailty, and diseases are needed.

In the subgroup analysis by gender, the effect of MEOHP on the risk of frailty was relatively greater in men than women, and the effect of MEHHP on the risk of frailty was relatively greater in women; the effect in men was statistically insignificant. This may be due to differences in the metabolism of DEHP between men and women. Certain exogenous substances, namely endocrine disruptor chemicals (EDCs), could interfere with androgen and estrogen effects differently for each gender. However, the reasons for the differential effects are as yet unclear [43]. Due to the relatively small sample size of men in this study, the findings need to be examined further in large-scale studies.

In our analysis of lifestyle (health behaviors) and DEHP, daily fruit intake was inversely associated with phthalate concentration level. This is consistent with existing evidence on nutrition and exposure to phthalates, which suggests diets high in fruits (and vegetables) are associated with a decrease in phthalate concentration [44,45,46]. In addition, the relationship between daily fruit consumption and frailty status was significantly negative in both males and females (see Supplementary Material Table S1 including the full Model 4 of Table 4); this is a finding consistent with García-Esquinas et al. (2016) and Teixeira-Gomes (2020) [25,47]. Older adults with fresh-food intake, including regular fruit intake, may have less instant food, which is typically wrapped in plastic, a typical source of phthalate exposure for (older) adults in daily living [48,49]. Vegetable consumption was not related in our sample, likely due to the relatively high level of vegetable consumption in the typical Korean diet among Korean older adults; for example, kimchi, a traditional Korean dish of salted and fermented vegetables, is served as a side with almost every meal [50]. Further studies are needed on the protective mechanism of a healthy diet including fruit intake on DEHP exposure. Health education along with service or monetary support programs (e.g., vouchers) to increase healthy nutrition for older adults is also recommended. The inverse relationship between smoking and DEHP metabolite level need to be cautiously interpreted as the majority of the study sample were women (68.5%) whose DEHP concentration is lower than men.

The purpose of this study was to increase methodological rigor in the investigation of phthalates and frailty. We defined and measured frailty with a composite measure of Fried’s five criteria [3], which are mostly well-known and widely accepted, rather than a single measure [20]. We also measured in detail the socioeconomic and health conditions of participants over time and adjusted for those multi-dimensional covariates in examining the relationship of interest. Panel data-based estimations of the relationship between phthalates and frailty can reduce the risk of reverse causality, but a large number of studies are cross-sectional, and only a few studies have reported evidence from a multi-year study [9,13]. This study addressed this evidence gap, though further studies are still necessary with larger sample sizes and in different places.

This study also has several limitations. The participants in the study were a convenient sample who were mainly recruited in community centers, which limits the ability to generalize the findings to the entire Korean older population. In spite of the panel-data approach, this study cannot show the causality of DEHP exposure and frailty. A detailed physiological pathway showing how DEHP affects the risk of being pre-frail or fail could not be investigated.

## 5. Conclusions

This study addressed the importance of environmental exposure—in particular, DEHP metabolites—to the functioning and frailty of an older population. Most environmental health studies have examined the direct health effects of environmental exposure on specific diseases in the populations of interest. The study findings suggest frailty can be a critical precursor or intermediate outcome in the path of how environmental exposures may prompt the onset or aggravation of certain diseases. Another interesting finding is that daily fruit intake can be a protective factor against environmental exposure and, in turn, the risk of frailty in older adults. Further studies are necessary on the interaction, process, and effects of exposures (risks) in complex, dynamic physical, and also social environments with regard to the function and health outcomes of older populations. Additional investigations are also needed on the profiles of highly susceptible people and protective factors in more diverse populations and contexts.

## Figures and Tables

**Table 1:** The measurement of frailty in the study.

Criterion ^1^	Item ^1^	Measure ^2^
Shrinking	Unintended weight loss	Score 1 point for participants who answer “Yes” to the question “In the last year, have you lost more than 5 kg unintentionally?”
Weakness	A decrease in the strength of handgrip: The lowest 20% of grip strength stratified for gender and body mass index (BMI) quartiles	Score 1 point for participants who meet any of the following conditions: Men, cut-off for grip strength BMI ≤ 21.4, cut-off criteria ≤22.1 kg BMI 21.4–23.3, cut-off criteria ≤25.0 kg BMI 23.3–25.2, cut-off criteria ≤26.5 kg BMI > 25.2, cut-off criteria ≤27.5 kg Women, cut-off for grip strength BMI ≤ 21.8, cut-off criteria ≤13.0 kg BMI 21.9–24.0, cut-off criteria ≤15.0 kg BMI 24.0–26.2, cut-off criteria ≤15.3 kg BMI > 26.2, cut-off criteria ≤15.4 kg
Poor endurance	Self-reported severe fatigue	Score 1 point for participants who answer “Yes” to either of the following statements from the two questions from the Center for Epidemiological Studies Depression scale: (a) I felt that everything I did was an effort (b) I could not get going
Slowness	A decrease in walking speed: The slowest 20% of walking speed over 2.5 m stratified for gender and height	Score 1 point for participants who meet any of the following conditions: Men, cut-off for time to walk 2.5 m Height ≤ 165.2 cm, cut-off criteria ≥5 s Height > 165.2 cm, cut-off criteria ≥4.5 s Women, cut-off for time to walk 2.5 m Height ≤ 153.0 cm, cut-off criteria ≥5.78 s Height > 153.0 cm, cut-off criteria ≥5.14 s
Low physical activity	A decrease in activity amount (kcal/week): The lowest 20% of energy expenditure estimates	Score 1 point for participants who meet any of the following conditions: Men: <494.65 kcal/week Women: <283.50 kcal/week

^1^ Adopted from Fried et al. [3]; ^2^ Adopted from Park et al. [23].

**Table 2:** General characteristics of the study population by frailty at baseline.

		Total	Fit	Non-Fit (Pre-Frail or Frail)	*p*-Value
		*n* (%)	*n* (%)	*n* (%)	
		767 (100.0)	324 (42.2)	443 (57.8)	
Socioeconomic Status					
Gender	male	242 (31.5)	100 (30.9)	142 (32.1)	0.726
	female	525 (68.5)	224 (69.1)	301 (67.9)	
Age	60–69	177 (23.1)	99 (30.7)	78 (17.6)	<0.001 ***
	70–79	448 (58.5)	197 (61.0)	251 (56.7)	
	80+	141 (18.4)	27 (8.3)	114 (25.7)	
Education	none	231 (30.1)	65 (20.1)	166 (37.5)	<0.001 ***
	elementary school	263 (34.3)	93 (28.7)	170 (38.4)	
	middle school+	273 (35.6)	166 (51.2)	107 (24.1)	
Living alone	yes	252 (32.9)	100 (30.9)	152 (34.3)	0.315
	no	515 (67.1)	224 (69.1)	291 (65.7)	
Household income	<1,000,000 KRW	375 (51.5)	133 (42.4)	242 (58.6)	<0.001 ***
(monthly)	≥1,000,000 KRW	111 (15.3)	65 (20.7)	46 (11.1)	
	don’t know	241 (33.2)	116 (36.9)	125 (30.3)	
Employment	yes	192 (25.0)	62 (19.1)	130 (29.3)	0.001 **
	no	575 (75.0)	262 (80.9)	313 (70.7)	
Location	urban	384 (50.1)	236 (72.8)	148 (33.4)	<0.001 ***
	rural	383 (49.9)	88 (27.2)	295 (66.6)	
Lifestyle					
Smoking status	yes	148 (19.3)	59 (18.2)	89 (20.1)	0.515
	no	619 (80.7)	265 (81.8)	354 (79.9)	
Drinking status	yes	226 (29.5)	99 (30.7)	127 (28.7)	0.553
	no	540 (70.5)	224 (69.3)	316 (71.3)	
Fruit consumption	every day	282 (36.8)	173 (53.4)	109 (24.6)	<0.001 ***
	not every day	485 (63.2)	151 (46.6)	334 (75.4)	
Vegetable consumption	every day	565 (73.7)	258 (79.6)	307 (69.3)	0.001 **
	not every day	202 (26.3)	66 (20.4)	136 (30.7)	
Health Conditions					
No. of drugs	0	420 (54.8)	158 (48.8)	262 (59.1)	0.003 **
	1	252 (32.9)	113 (34.9)	139 (31.4)	
	2+	95 (12.3)	53 (16.3)	42 (9.5)	
BMI	<23	308 (40.2)	99 (30.6)	209 (47.2)	<0.001 ***
	23–25	206 (26.8)	98 (30.3)	108 (24.4)	
	≥25	253 (33.0)	127 (39.2)	126 (28.4)	
No. of chronic diseases	0	205 (26.8)	103 (31.8)	102 (23.1)	0.009 **
	1	334 (43.6)	140 (43.2)	194 (43.9)	
	2+	227 (29.6)	81 (25.0)	146 (33.0)	

Abbreviations: KRW = South Korean Won; ** *p* < 0.01, *** *p* < 0.001.

**Table 3:** Di-ethylhexyl phthalate (DEHP) concentration by frailty at baseline.

		Total			Fit			Non-Fit (Pre-Frail or Frail)			*p*
		*n*	GM (μg/L)	SE	*n*	GM (μg/L)	SE	*n*	GM (μg/L)	SE	
MEHHP	Total	767	28.06	0.85	324	22.40	0.92	443	33.08	1.00	<0.001 ***
	Q1	191	10.01	0.46	112	10.31	0.52	79	9.60	0.80	<0.001 ***
	Q2	192	22.58	0.24	97	22.42	0.34	95	22.73	0.33	
	Q3	192	36.09	0.36	70	35.30	0.57	122	36.54	0.47	
	Q4	192	75.58	2.45	45	75.87	6.61	147	75.49	2.51	
MEOHP	Total	767	21.39	0.58	324	17.52	0.82	443	24.75	0.85	<0.001 ***
	Q1	191	8.50	0.27	112	8.55	0.34	79	8.44	0.44	<0.001 ***
	Q2	192	16.89	0.15	86	16.82	0.21	106	16.94	0.21	
	Q3	192	26.92	0.27	83	26.49	0.42	109	27.25	0.34	
	Q4	192	53.89	1.73	43	55.51	4.77	149	53.42	1.78	

Abbreviations: GM = geometric mean; SE = standard error; MEHHP = Mono (2-ethyl-5-hydroxyhexyl) phthalate MEOHP = Mono(2-ethyl-5-oxohexyl) phthalate; *** *p* < 0.001.

**Table 4:** Association between log-transformed DEHP concentration and frailty (multivariate, multi-year analysis).

	Frailty [Outcome= Non-Fit (Pre-Frail or Frail)]							
	Model 1		Model 2		Model 3		Model 4	
	OR (CI)	QIC	OR (CI)	QIC	OR (CI)	QIC	OR (CI)	QIC
Total								
log-MEHHP	1.58 *** (1.38, 1.81)	3045.4	1.27 ** (1.10, 1.47)	2613.4	1.24 ** (1.07, 1.44)	2574.5	1.25 ** (1.07, 1.46)	2559.8
log-MEOHP	1.51 *** (1.34, 1.71)	3052.3	1.26 *** (1.10, 1.45)	2613.2	1.24 ** (1.08, 1.43)	2573.9	1.25 ** (1.08, 1.44)	2559.4
Male								
log-MEHHP	1.33 * (1.06, 1.69)	887.8	1.18 (0.93, 1.50)	787.5	1.16 (0.90, 1.48)	776.9	1.18 (0.91, 1.52)	779.7
log-MEOHP	1.43 ** (1.14, 1.79)	881.7	1.29 * (1.01, 1.66)	785.3	1.28 (1.00, 1.65)	775.1	1.33 * (1.02, 1.73)	777.6
Female								
log-MEHHP	1.76 *** (1.50, 2.06)	2152.3	1.31 ** (1.09, 1.57)	1822.5	1.28 * (1.06, 1.55)	1797.7	1.28 * (1.06, 1.55)	1791.7
log-MEOHP	1.56 *** (1.35, 1.80)	2173.6	1.24 * (1.04, 1.46)	1825.7	1.21 * (1.02, 1.44)	1800.6	1.20 * (1.01, 1.43)	1794.8

Model 1: unadjusted; Model 2: adjusted for socioeconomic status (SES); Model 3: adjusted for SES and lifestyle; Model 4: adjusted for SES, lifestyle, and health conditions; * *p* < 0.05, ** *p* < 0.01, *** *p* < 0.001.

**Table 5:** Lifestyle factors related to DEHP concentration (multivariate, multi-year analysis ^1^).

Lifestyle		log-MEHHP	log-MEOHP
		β (SE)	β (SE)
Total			
Smoking (ref.= no)	yes	−0.09 (0.05)	−0.09 (0.05)
Drinking (ref.= no)	yes	−0.03 (0.04)	−0.05 (0.04)
Fruit consumption (ref.= no)	yes	−0.11 (0.03) **	−0.10 (0.03) **
Vegetable consumption (ref.= no)	yes	0.01 (0.04)	0.00 (0.04)
Male			
Smoking (ref.= no)	yes	−0.04 (0.06)	−0.05 (0.06)
Drinking (ref.= no)	yes	−0.06 (0.07)	−0.1 (0.06)
Fruit consumption (ref.= no)	yes	−0.14 (0.07) *	−0.12 (0.06)
Vegetable consumption (ref.= no)	yes	−0.06 (0.08)	−0.05 (0.07)
Female			
Smoking (ref.= no)	yes	−0.22 (0.1) *	−0.15 (0.10)
Drinking (ref.= no)	yes	−0.01 (0.04)	−0.02 (0.05)
Fruit consumption (ref.= no)	yes	−0.10 (0.04) **	−0.09 (0.04) *
Vegetable consumption (ref.= no)	yes	0.03 (0.04)	0.02 (0.04)

^1^ All socioeconomic and health condition covariates in Table 1 were adjusted for; * *p* < 0.05, ** *p* < 0.01.

## Data Availability

No new data were created or analyzed in this study. Data sharing is not applicable to this article.

## Supplementary Materials

The following are available online at https://www.mdpi.com/1660-4601/18/4/1985/s1, Table S1: Factors associated with frailty by gender.
