# Supplementary material for: Association between Phthalate Exposure and Frailty among Community-Dwelling Older Adults: A Repeated Panel Data Study
Source: Int J Environ Res Public Health. 2021 Feb 18;18(4):1985. doi: 10.3390/ijerph18041985 (PMC7922338; doi:10.3390/ijerph18041985)
Supplement: Supplementary file 1 [file ijerph-18-01985-s001.pdf]

Supplementary Material

Table S1. Factors associated with frailty by gender [1].

		Frailty [Outcome= Non-Fit (Pre-Frail or Frail)]											
		Male			Female			Male			Female		
		OR	CI		OR	CI		OR	CI		OR	CI	
Socioeconomic status													
Age (ref.= 60–69)	70–79	1.65	0.98	2.77	0.99	0.71	1.37	1.67	0.99	2.80	0.99	0.72	1.38
	80+	2.78 ***	1.49	5.18	2.61 ***	1.66	4.10	2.79 **	1.49	5.20	2.65 ***	1.69	4.16
Education	none	2.83 **	1.48	5.39	2.29 ***	1.63	3.23	2.85 **	1.49	5.46	2.28 ***	1.62	3.21
(ref.= middle school)	elementary school	1.88 **	1.20	2.94	1.71 **	1.20	2.43	1.89 **	1.21	2.96	1.70 **	1.20	2.41
Living alone (ref.= no)	yes	1.39	0.81	2.38	1.24	0.94	1.63	1.38	0.80	2.36	1.24	0.94	1.63
Household income monthly	≥1,000,000 KRW	0.62 *	0.41	0.94	0.76	0.53	1.10	0.59 *	0.39	0.90	0.76	0.53	1.10
(ref. < 1,000,000 KRW)	don’t know	0.90	0.54	1.51	0.89	0.68	1.19	0.90	0.54	1.51	0.90	0.68	1.19
Employment (ref.= no)	yes	0.93	0.60	1.45	1.13	0.81	1.58	0.93	0.60	1.46	1.13	0.81	1.57
Location (ref.= urban)	rural	1.42	0.86	2.34	2.85 ***	2.06	3.94	1.44	0.87	2.37	2.74 ***	1.97	3.80
Lifestyle													
Smoking status (ref.= no)	yes	1.05	0.73	1.51	1.16	0.56	2.39	1.04	0.72	1.49	1.19	0.57	2.48
Drinking status (ref.= no)	yes	1.22	0.84	1.77	0.99	0.73	1.35	1.20	0.83	1.75	0.99	0.73	1.35
Fruits consumption (ref.= everyday)	not everyday	1.27	0.87	1.85	1.75 ***	1.37	2.25	1.29	0.88	1.87	1.74 ***	1.36	2.23
Vegetable consumption (ref.= everyday)	not everyday	1.83 **	1.18	2.85	1.03	0.77	1.38	1.85 **	1.19	2.86	1.04	0.78	1.39
Health conditions													
No. of medications (ref.= 0)	1	0.70	0.47	1.06	1.01	0.78	1.30	0.71	0.47	1.07	1.01	0.78	1.30
	2+	0.75	0.42	1.35	0.97	0.69	1.37	0.75	0.42	1.34	0.97	0.69	1.37
BMI (ref. < 23)	23–25	0.74	0.46	1.19	0.84	0.60	1.16	0.75	0.47	1.20	0.83	0.60	1.15
	≥25	0.50 **	0.30	0.83	0.67 **	0.50	0.90	0.51 *	0.31	0.85	0.66 **	0.49	0.88
No. of chronic diseases (ref.= 0)	1	1.10	0.73	1.65	1.27	0.93	1.75	1.10	0.73	1.65	1.27	0.92	1.74
	2+	1.51	0.94	2.43	1.74 **	1.25	2.42	1.48	0.92	2.38	1.72 **	1.24	2.40
Phthalate concentration													
Log-MEHHP		1.18	0.91	1.52	1.28 *	1.06	1.55						
Log-MEOHP								1.33*	1.02	1.73	1.20 *	1.01	1.43

[1] These are the full results of Model 4 by gender from Table 4 in the manuscript; Abbreviations: KRW = South Korean Won; * $p < 0.05$, ** $p < 0.01$, *** $p < 0.001$.
